# Diversity, evolution, and classification of virophages uncovered through global metagenomics

**DOI:** 10.1186/s40168-019-0768-5

**Published:** 2019-12-10

**Authors:** David Paez-Espino, Jinglie Zhou, Simon Roux, Stephen Nayfach, Georgios A. Pavlopoulos, Frederik Schulz, Katherine D. McMahon, David Walsh, Tanja Woyke, Natalia N. Ivanova, Emiley A. Eloe-Fadrosh, Susannah G. Tringe, Nikos C. Kyrpides

**Affiliations:** 1 Department of Energy, Joint Genome Institute, 2800 Mitchell Dr., Walnut Creek, 94598 USA; 2 BSRC “Alexander Fleming”, 34 Fleming Street, Vari, 16672 Athens, Greece; 3 Departments of Civil and Environmental Engineering and Bacteriology, University of Wisconsin Madison, 1550 Linden Drive, Madison, WI 53726 USA; 4 Department of Biology, Concordia University, 7141 Sherbrooke St. West, Montreal, QC, H4B 1R6 Canada

**Keywords:** Metagenomics, Virophage, Major capsid protein (MCP), Virophage classification, Virophage-NCLDV interactions, Global distribution

## Abstract

**Background:**

Virophages are small viruses with double-stranded DNA genomes that replicate along with giant viruses and co-infect eukaryotic cells. Due to the paucity of virophage reference genomes, a collective understanding of the global virophage diversity, distribution, and evolution is lacking.

**Results:**

Here we screened a public collection of over 14,000 metagenomes using the virophage-specific major capsid protein (MCP) as “bait.” We identified 44,221 assembled virophage sequences, of which 328 represent high-quality (complete or near-complete) genomes from diverse habitats including the human gut, plant rhizosphere, and terrestrial subsurface. Comparative genomic analysis confirmed the presence of four core genes in a conserved block. We used these genes to establish a revised virophage classification including 27 clades with consistent genome length, gene content, and habitat distribution. Moreover, for eight high-quality virophage genomes, we computationally predicted putative eukaryotic virus hosts.

**Conclusion:**

Overall, our approach has increased the number of known virophage genomes by 10-fold and revealed patterns of genome evolution and global virophage distribution. We anticipate that the expanded diversity presented here will provide the backbone for further virophage studies.

## Background

Virophages are a group of circular double-stranded DNA (dsDNA) viruses taxonomically classified within the *Lavidaviridae* family [1]. They co-infect unicellular eukaryotic hosts with members of the *Mimiviridae* family, a group of nucleocytoplasmic large DNA viruses (NCLDV) [2–4]. By siphoning off resources within the giant virus factory, virophage replication reduces the number of giant virus progeny, thereby increasing host survival [5].

Since 2008, when virophages were discovered in a water-cooling tower (virophage *Sputnik*) [5], genome sequences have been obtained for five cultured isolates: *Sputnik2* (from lens liquid), *Sputnik3* (from soil), *Mavirus* (from coastal waters), *Zamilon* (from soil), and *Zamilon2* (from a bioreactor) [5–9]. These five virophages have been classified into two genera: *Sputnikvirus* (including *Sputnik* and *Zamilon* genomes) and *Mavirus* [10]. All five reference isolated genomes lack an envelope, form small icosahedral capsids (diameter of 35–74 nm), and have genomes ranging from 17 to 19 kb in length [11].

The rate of discovery of new virophages recently took a big leap due to the recovery of 20 virophage genomes from metagenomes. Of these, 18 were identified in diverse lake microbiomes (from Antarctica [12, 13], China [14, 15], and the US [13, 16, 17]) and the remaining two were assembled from sheep rumen samples [18]. Additionally, partial virophage genome sequences have been detected in these same samples and in various aquatic environments (marine water, wastewater, sludge [13, 18]) as well as in non-aquatic habitats (soils, air, bioreactors, animal, or human gut). Although mammals could be exposed to giant viruses and virophages, and giant viruses have been isolated from human fecal and lung samples [19–21], there is very limited evidence of virophages being present in humans [11].

Virophage genomes display highly variable gene content and are most closely related to members of polintons (a widespread group of eukaryotic large DNA transposons [22]). Only four genes are conserved in almost all known virophage genomes: (1) MCP and (2) mCP, major and minor capsid proteins, respectively, involved in morphogenesis; (3) an ATPase involved in DNA packaging; and (4) PRO, a cysteine protease implicated in capsid maturation [16, 18]. Among these “core” genes, MCP sequences have been used as bait for the discovery of new virophage genomes [18] since the ATPase and PRO genes have homologs outside the virophage group, and the mCP was not always detected with stringent search criteria.

Here, we generated new hidden Markov models (HMMs) for virophage MCPs through a two-step process and used these HMMs to search for virophage genomes in 14,000 publicly available microbiomes from ecologically diverse samples. This resulted in the identification of 328 diverse new virophage genomes containing all four core genes, which led to a major revision of the classification of the *Lavidaviridae* (virophage) family. Finally, we computationally predicted putative associated giant viruses for a subset of virophages.

## Results

### Vast diversity and global distribution of virophage major capsid proteins (MCPs) across microbiomes

**Fig. 1:**
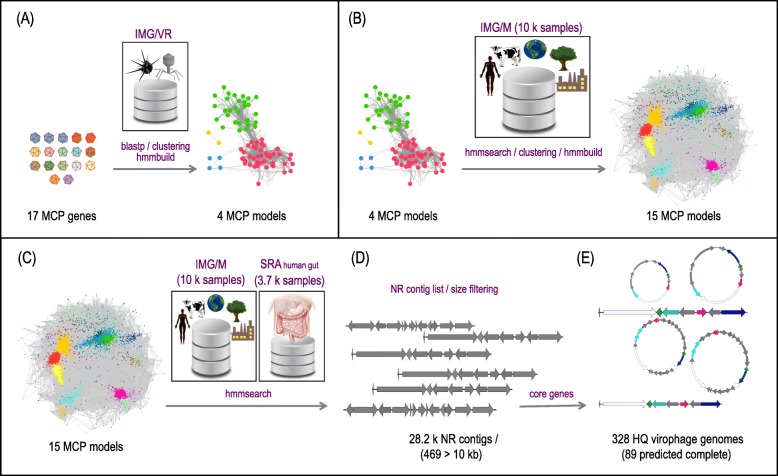
Virophage discovery pipeline. **a** MCP amino acid sequences from reference isolated genomes and published metagenomic contigs were queried against the IMG/VR database with stringent *e* value cutoffs. All homologous sequences detected were then clustered together to build four independent MCP profiles (HMM models; see details in the “Methods” section). **b** The resulting four MCP models were used to recruit additional homologous sequences from the entire IMG/M system (containing over 10,000 public samples). All new sequences were clustered, and models were built creating a final set of 15 unique MCP HMMs. **c** These 15 unique MCP HMMs were then used to search two different databases for homologous sequences: the IMG/M system and a custom assembled human gut database containing 3771 samples from NCBI’s Sequence Read Archive (SRA). **d** The resulting set of 28,294 non-redundant (NR) sequences (Additional file 1: Table S1) with stringent *e* value cutoffs was filtered by size and **e** by the presence of the four core virophage genes (high-quality genomes; HQ virophages). Finally, we predicted completeness of novel metagenomic virophage genomes based on circularity or presence of inverted terminal repeats (ITR)

Virophages have been previously detected from microbiome datasets using the major capsid protein (MCP) genes as signature sequences in homology-based queries [6, 12–15, 17, 18, 23]. Here, we combined known MCP sequences with homologous sequences recruited from the Integrated Microbial Genomes with Viruses database (IMG/VR) [24] and over 10,300 diverse microbiomes from the Integrated Microbial Genomes with Microbiomes (IMG/M) system [25] to generate 15 new virophage MCP hidden Markov models (details in the “Methods” section). These models were then used as bait to capture new virophage sequences from a large set of geographically and ecologically diverse samples that included all of the public IMG/M microbiomes together with an assembled set of 3771 human gut datasets downloaded from the NCBI’s Sequence Read Archive (SRA) [26] (see the “Methods” section and Fig. 1).

**Fig. 2:**
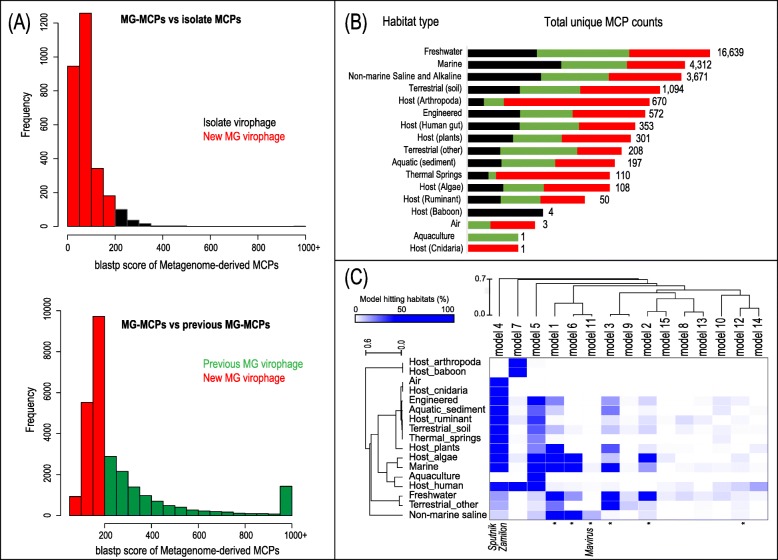
Diversity and habitat distribution of MCP sequences. **a** MCP sequence diversity of the 28,294 non-redundant sequences (de-replicated at 95% identity over 95% of the shortest length; see the “Methods” section) represented by a blastp score histogram against isolate virophage MCPs (upper) or previously reported metagenome-derived MCPs (bottom). The more dissimilar MCP sequences (score < 200) are shown in red while those related to MCPs from isolated virophages (*Sputnik*, *Mavirus*, and Zamilon) or previously published MCP sequence are shown in black and green, respectively. **b** Habitat type distribution of the non-redundant MCP dataset. Total number of MCP counts by habitat type in logarithmic scale. Colors represent the proportion (non-logarithmic) of non-redundant MCP sequences from the groups in panel **a**; code: MCP counts from similar to an isolated virophage in black; MCP counts from similar to a previously published virophage in green; MCP counts from more dissimilar detected sequences in red. **c** Link between MCP models and the habitat types where their associated sequences were found. The heat map indicates the percentage of hits to each MCP model per habitat type. MCP models containing sequences from isolated virophages or reference metagenomes are indicated at the bottom with the name of the isolate or with an asterisk, respectively. Hierarchical clustering (complete linkage) of both models and habitats was applied after a quantile normalization. Although unlikely, some MCP sequences identified on short contigs with uncertain origin may derive from virophage MCPs integrated in their host genomes

This approach led to the identification of 44,221 total virophage sequences (Additional file 1: Table S1), including 28,294 new non-redundant MCP sequences (4% of them predicted as complete or near-complete genes; the “Methods” section) that were compared against the isolate virophage MCPs and the previously published metagenomic MCPs to build two histograms that reflect the breadth of this gene sequence space (Fig. 2a), greatly expanding the known diversity of this virophage marker. Most of the non-redundant newly discovered MCP sequences (88%) were found in aquatic environments (including freshwater and marine samples) (Fig. 2b). This was expected due to a fair representation of these habitat types (11% and 15% of freshwater and marine samples, respectively) in the public databases (details in the “Methods” section) [27, 28] and especially in the published genomes from which the MCP models were generated. The remaining 12% of MCPs were found in diverse habitats including different types of soil, distinct host-associated microbiomes, and various bioreactor samples (Fig. 2b). Each MCP model typically retrieved virophage sequences from multiple habitats; for example, MCP models four and five targeted virophage sequences from several habitats, although they were predominantly found in freshwater and marine systems, respectively. However, some models only retrieved sequences from specific habitats, e.g., MCP models 1, 2, 6, 11, and 15 were found almost exclusively in aquatic samples and models 7, 13, and 14 were only associated with arthropods, ruminants, or human gut-associated samples (Additional file 1: Table S2; Fig. 2c). The two-step iterative process enabled a deeper search establishing associations between the MCP models and novel habitat types. When habitat types were clustered based on the fraction of hits from any model, two separate habitat groups were observed: habitats where virophages were previously undetected (predominantly host-associated, including samples from human, baboon, and arthropods; air; sediments and engineered microbiomes), and those where the presence of virophages was previously known (i.e., aquatic and terrestrial microbiomes) (Fig. 2c). A multi-model approach with iterative model refinement is thus highly valuable for discovering new members of virus groups with only a handful of references.

### Virophage genome recovery and completeness

Four hundred sixty-nine non-redundant metagenomic virophage sequences were larger than 10 kb (Additional file 1: Table S3) and were selected for further analysis together with 58 published virophage and related sequences (33 complete and near-complete virophages and 25 polinton viruses). Protein coding genes from those contigs were extracted and grouped into families using a two-step approach, which generated a set of 711 virophage protein clusters (VpPCs) (see the “Methods” section and Additional file 1: Table S4). Using a combination of filtering criteria that included the presence of the four core virophage genes (MCP, mCP, ATPase, and PRO), a minimum contig size of 10 kb, and sequence de-replication, we identified 328 virophage contigs as “near-complete” which will be referred to hereafter as high quality (HQ) (Fig. 1e). Of those contigs, 89 were likely to represent complete genomes based on additional features such as predicted circularity and/or the presence of inverted terminal repeats (ITR) (51 circular, 35 ITR, and 3 with both features). Prior to our study, the complete genomes from 23 predicted virophages ranged in size from 13.8 to 29.7 kb and encoded 13 to 25 genes [11, 13, 16]. The newly identified 89 complete virophages expanded the putative genome size range from 10.9 to 42.3 kb and the range of gene counts from 12 to 39 (Additional file 1: Table S5). Interestingly, the mCP, a penton protein homolog that displays a single jelly-roll fold [29], was split into two separate VpPCs. One of these was exclusively identified in rumen and human microbiome samples and carried a distinct sequence pattern, which could explain why it remained unrecognized as an mCP in previous analyses of rumen samples [18].

The 328 HQ virophage genomes were distributed across differing ecosystems (freshwater, marine, engineered, host-associated, soils, and thermal spring samples) and a wide variety of geographical and ecological niches. For example, within the freshwater habitat type, we recovered HQ virophage genomes from wetlands and freshwater sediments, as well as lakes in northern Canada, midwestern USA (Wisconsin, North Dakota, Minnesota, Ohio, and Kansas), southeastern USA (Georgia), California (Yosemite), Germany, and Congo (Additional file 1: Table S5). We also recovered HQ virophage genomes from multiple marine habitats ranging from coastal waters to deep-ocean and hydrothermal vents, across different types of soils, and in a great variety of host-associated samples including plants, ruminants, and humans (Additional file 1: Table S5).

### Expanding the virophage classification

**Fig. 3:**
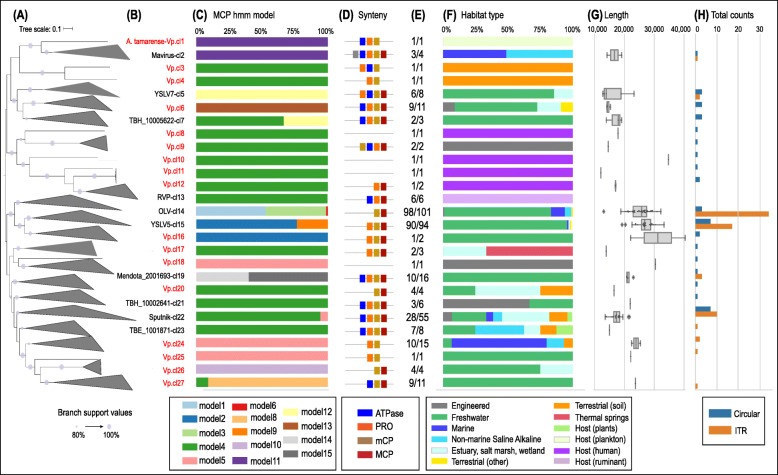
Classification scheme of virophage genomes and details of new clades. **a** Maximum likelihood phylogenetic tree of HQ virophage genomes, i.e., virophage sequences > 10 kb containing the four core genes, based on the concatenated alignment of MCP, mCP, ATPase, and PRO. Each of the 27 clades corresponds to a proposed clade. The scale bar represents substitutions per site. Branch support values are shown at each node and indicated in the legend. **b** Virophage clade (Vp.cl) identifier. Names of clades containing previously reported virophage genomes include the name of one known virophage (e.g., OLV-cl14). Names of the new clades are shown in red. **c** The distribution of MCP models best hit in the virophage clade. **d** Synteny structure of the four core virophage genes of the HQ virophage genomes. **e** The proportion of the members of each clade with the syntenic organization shown in **d**. **f** Habitat types of all the sequences in a clade with their color code description in the captions below. **g** Genome length distribution of the virophage genomes per clade. **h** Total number of virophage genomes with circular genomes (blue) or linear genomes with inverted terminal repeats (orange) per virophage clade. Note that **g** and **h** only display the information for the 89 complete virophage genomes

In order to infer the phylogenetic relationships of the newly identified 328 HQ virophage genomes to the published virophages, a phylogenetic tree was constructed based on the concatenated alignment of the four core genes (full-length) (see the “Methods” section). We identified 27 distinct well-supported clades (Vp.cl), 17 of which (comprising 64 sequences) had no published sequences and are thus considered novel (Fig. 3a, b). The remaining 10 groups containing published genome representatives were also greatly expanded through the addition of 264 sequences corresponding to a ~ 9-fold increase over the previously known published sequences. The expansion of the previously characterized clades was observed even in some of the best-represented groups. For example, Vp.cl14 (containing the OLV, QLV, DSLV1, YSLV1, YSLV2, YSLV3, YSLV4, YSLV6, and seven different Lake Mendota virophages) and Vp.cl15 (containing YSLV5, *Bigelowiella natans* virophage and 3 Trout Bog virophages) were expanded by 87 and 90 new members, respectively. A strong correlation was observed between members of each clade in terms of organization of the four core genes along the genome, habitat type, and closest MCP model (Fig. 3c–f). For example, 9 of 11 members of Vp.cl27 had the ATPase, mCP, and MCP genes colocalized (in this order), 11 members were found in freshwater habitats, and 10 of the MCP genes were detected via the HMM model #8. Genome length within a clade tended to be homogeneous, except for clades 5 and 16 where a twofold variation in genome length was observed (Fig. 3g). No correlation between predicted genome structure (circularity vs. ITR) and clade affiliation was observed (Fig. 3h). The previously reported rumen virophage sequences [18] were clustered within the same clade (Vp.cl13) along with four new sequences from the same habitat type.

### Conserved syntenic regions among virophage genomes

A colocalization of the four core genes (MCP, mCP, PRO, and ATPase) plus an additional gene putatively coding for a methyltransferase (MTase) was previously detected only in *Mavirus* and its close relative *Acid Lake Mavirus* virophage (ALM), but here was also observed in 18 of the 328 HQ virophage genomes (5.5%) (Additional file 2: Figure S1). A truncated version of this gene cluster with only four core genes was also detected in 64 of the remaining novel virophages, though nine displayed some change in their order. Both the four- and five-gene versions of the cluster were sporadically distributed across the HQ virophage tree (Additional file 2: Figure S1), suggesting this gene block was likely inherited from the common ancestor of all virophages. The gene synteny was further truncated to three core genes without ATPase in an additional 95 newly identified HQ virophages of distinct lineages (Additional file 2: Figure S1). One hundred seventy-six of the 328 HQ virophages contained adjacent ATPase, mCP, and MCP and 295 retained adjacent MCP and mCP genes despite undergoing multiple apparent re-organizations (Additional file 2: Figure S1), further confirming the strong linkage between these two capsid core genes. Another conserved syntenic gene cluster encoding a retroviral integrase (rve-INT) and DNA polymerase type B (DNApolB) was previously only identified from *Mavirus* and ALM but was found in six new HQ virophage genomes spread among distinct clades of virophages (Additional file 2: Figure S1). Phylogenetic trees of these two genes confidently grouped them with two polintons from *Polysphondylium pallidum* PN500 and *Dictyostelium lacteum* (branch labelled in red in Additional file 2: Figure S2), confirming the common origin of these genes and suggesting an ancestral gene exchange of the rve-INT and DNApolB gene module between polintons and virophages (Additional file 2: Figure S2).

### Virophage gene repertoire

The VpPCs computed from all virophage sequences ≥ 10 kb were classified into three groups: (1) the four core gene families present in all HQ virophage genomes; (2) the common gene families, defined as being present in 25–60% of the virophage genomes, which included only 8 VpPCs (1.25%); and (3) the accessory families (98.0% of all VpPCs), defined as those detected in less than 25% of all the predicted virophage genomes (Additional file 2: Figure S3). Common VpPCs could be associated with a predicted function, e.g., VpPC_007 (site-specific DNA adenine methylase), VpPC_005 (phage integrase/recombinase), and VpPC_012 (phage DNA primase/helicase) (see details in Additional file 2 and Additional file 1: Table S6).

We investigated the presence of VpPCs across the different virophage clades and observed 13 clusters present in more than 30% of them (Additional file 2: Figure S4; Additional file 1: Table S4 and Table S7). In contrast, 87 VpPCs were found in only one clade, suggesting these could be considered marker genes for these groups (Additional file 2: Figure S4; Additional file 1: Table S6 and Table S7). Interestingly, when virophage clades were clustered based on the total VpPC content of their members, the resulting groups agreed with the phylogeny inferred from the concatenated four core genes (Additional file 2: Figure S4). Clades composed mainly of members from freshwater environments grouped together, as well as clades containing members from marine or wetland habitats. Similarly, the two clades with terrestrial virophages clustered together, and so did the clades comprising human-gut and ruminant virophages. We also divided the HQ virophages by habitat to investigate the presence of habitat-specific marker VpPCs (Additional file 2: Figure S5; Additional file 1: Table S6 and Table S8) and discovered the presence of hypothetical proteins exclusively found in marine virophages, as well as other proteins exclusively present in both rumen and human habitats (Additional file 2: Figure S5; Additional file 1: Table S6 and Table S8). These observations reveal that despite the considerable shuffling of virophage genomes and diversity of gene content, there are also clear group-specific and habitat-derived patterns in the genetic content of the different virophage clades.

Transfer ribonucleic acid sequences (tRNAs) were encoded in 18 HQ virophage genomes (Additional file 2: Figure S6; Additional file 1: Table S9) as well as in 12 additional virophage sequences (> 10 kb but without all four core genes present). Although the presence of tRNAs is not unusual for phage genomes (found in 7% and 7.6% of reference isolate viruses and metagenomic viral contigs, respectively [30]), this is the first time that these genes have been noted in virophages. tRNA sequences were identified in HQ virophage contigs from clades 4, 14, 15, and 22 (Additional file 2: Figure S6). These tRNA sequences did not display high sequence similarity to any tRNAs in isolate genomes in NCBI or IMG databases, and therefore, their origin is uncertain. Interestingly, although the genome composition of the tRNA-encoding virophages was extremely diverse, 57% of the clade 14 tRNAs recognized methionine (all CAT anticodons) and 87.5% of the clade 15 tRNAs recognized glutamine (6 TTG and 1 CTG anticodons). The remaining tRNAs recognized leucine (clade 14, anticodon TAA; clade 15, anticodon TAA), proline (clade 22, anticodon TGG), cysteine (clade 22, anticodon ACA), phenylalanine (clade 4, anticodon AAA), and an ochre stop codon (clade 22, anticodon TTA) (Additional file 2: Figure S6). The presence of an ochre-specific tRNA may be indicative of stop codon reassignment in the hosts of these virophages [31]. As in other viruses, the presence of these tRNAs could complement their host’s codon or amino acid usage [32, 33] or could be a result of an acquisition from the host genome, since tRNAs are known as hot spots for virus integration [32, 34, 35]. To support the latter hypothesis, we observed that all the complete virophage genomes with tRNA sequences (seven genomes from two clades) contained a predicted integrase gene (VpPC_005) suggesting that these virophages could have been integrated into their host’s genome.

### Recovery of virophages from human gut samples

**Fig. 4:**
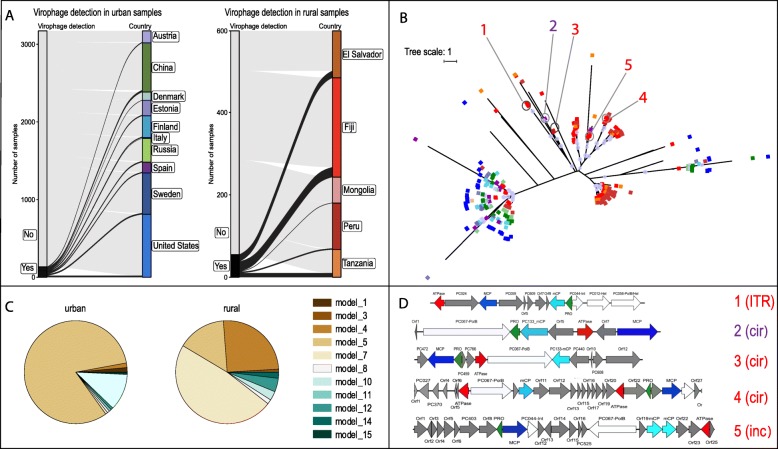
Human gut-associated virophage sequences. **a** Geographic and lifestyle distribution of the human gut samples queried for the presence of virophages. Gray denotes samples with no hits to any of the MCP models, while black colors correspond to samples with hits to different MCP models from the indicated country. **b** Unrooted maximum likelihood phylogenetic tree of the 353 MCP sequences detected in the human gut samples. Branch support values > 90% are shown at each node using purple circles. Colored squares at the tip of the branches indicates the country of the sample according to the color code of panel **a**: “warm colors” (red, brown, orange, amber) or “cold colors” (blues, greens, and purples) represent samples from countries with rural or westernized lifestyles according to sample metadata, respectively. MCP genes found in sequences longer than 10 kb are indicated with numbers 1–5 and colored according to the country where they were detected. **c** Proportion of the MCP sequences detected by different HMM models (corresponding to different colors as indicated) in westernized and rural lifestyles. **d** Genetic organization of the 5 gut virophage genomes longer than 10 kb. The four core genes were colored as follows: red denotes ATPase, dark blue MCP, light blue mCP, and green PRO. Other common genes (in white) or unknown genes (in gray) are also displayed and their protein cluster (PC) or annotation indicated when possible (Int, integrase; Hel, helicase; PolB, polymerase B). Numbers 1–5 and their colors correspond to the same numbers and sample colors shown in panel **b**. 1, SRS475626|k119_215568 (17,831 bp; clade 8); 2, ERS396424|k79_177141 (12,062 bp; clade 11); 3, SRS476271|k119_132073 (17,103; clade 12); 4, SRS476076|k119_199462 (34,763 bp; clade 10); 5, SRS476192|k119_38656 (31,481 bp; clade 12). The circularity (cir) or the incompleteness of the genome (inc), as well as the presence of an inverted terminal repeat (ITR), are indicated next to the number

A total of 353 virophage sequences (five of them HQ genomes) were newly identified across 247 human gut microbiome samples, all of which were from the human gut datasets assembled from the SRA records [26]. This is the first report of HQ virophages in human samples. A detailed manual review of sample metadata, including patient gender, lifestyle, age, body mass index (BMI), health condition, and country of origin, revealed a strong association between the presence of human-gut virophage sequences and a lifestyle classified as “rural” (e.g., hunter gatherers, traditional agriculturalists, villagers, and subsistence farmers). Specifically, we found that 65% of the putative human-gut virophage sequences were identified in samples from individuals associated with a rural lifestyle although these individuals only accounted for 15% of the total human fecal samples (Fig. 4a). Further, based on the maximum-likelihood phylogenetic tree, we found that virophage MCPs clustered according to host lifestyle, with those from rural and westernized samples forming distinct clades (Fig. 4b). This trend was also supported by the fact that virophage sequences from individuals in westernized and rural lifestyles were identified by different MCP HMMs; specifically, model #5 accounted for ~ 82% of the virophage sequences detected in westernized lifestyle samples as opposed to models #4 and #7, that together accounted for ~ 75% of sequences detected in the rural samples (Fig. 4c). Of the 353 human gut-associated virophage genomes, only five were longer than 10 kb (ranging from 12 kb to 34.7 kb), four of which were predicted to be complete based on circularity or ITR (Fig. 4d). Although the MCP genes from these five genomes were captured by the HMM model #4, they shared < 25% amino acid identity over 20% of the shortest sequence length (Fig. 4b) and were classified into different clades 8, 10, 11, and 12 according to the four core gene classification scheme. The genetic repertoire of these putative virophages varied greatly and displayed a large number of genes encoding for hypothetical or unknown functions. Interestingly, all of these five genomes contained a polinton-type DNA polymerase (PolB) (encoded either by VpPC_067 or VpPC_056), suggesting they are virophage-polinton hybrids similar to the recently described rumen virophages [18]. Additionally, human-associated and rumen virophages carried a distinct sequence pattern for the mCP (VpPC_133), so far exclusively identified in these habitats.

### Computational prediction of virophage hosts

The 33 previously known virophages have been shown or predicted to co-infect unicellular eukaryotes (amoebas, protozoans, and microalgae) with members of the *Mimiviridae* family of giant viruses [11, 16]. Experimentally, only Sputnik (co-infecting with a *Mamavirus* or a *Lentillevirus*), *Mavirus* (co-infecting with a *Cafeteria roenbergensis* virus (CroV)), and *Zamilon* (co-infecting with a *Mont1 Mimivirus*) have been associated with their eukaryotic hosts *C. roenbergensis* (*Mavirus*) or *Acanthamoeba polyphaga* (*Sputnik* and *Zamilon*). Computational approaches have previously been used to predict virophage co-infecting with giant viruses based on co-occurrence [16] and found putative co-infecting NCLDVs for 19 virophages, all members of the *Mimiviridae*, as well as three putative associations with different protozoan hosts.

**Fig. 5:**
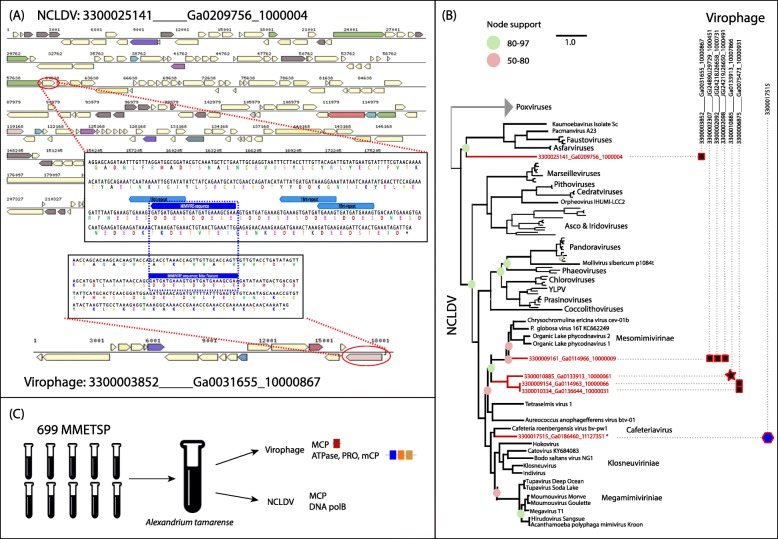
Computational prediction of virophage hosts. **a** Example of the MIMIVIRE system between a virophage contig (bottom; taxon and scaffold identifiers from the IMG/M system = 3300003852_____Ga0031655_10000867) and a NCLDV (up; taxon and scaffold identifiers from the IMG/M system = 3300025141_____Ga0209756_1000004). Both viral sequences shared a common 27-nt pattern that encodes two DDES peptidic motifs (highlighted in dark blue and in a blue box). In addition, the NCLDV genome has three copies of an 18-nt substring of the same sequence with the same motifs (in light blue). **b** Phylogeny of nucleocytoplasmic large DNA viruses (NCLDV) predicted as hosts of metagenomic virophages. Phylogenetic tree (IQ-tree LG + F + R6) of NCLDV inferred from a concatenated protein alignment of five core nucleocytoplasmic virus orthologous genes [38]. The tree was built from a representative set of NCDLV genomes after de-replication by ANI clustering (95% id). Red squares indicate virophage-host linkage as predicted by the MIMIVIRE system, red star shows the linkage of virophage-host by MIMIVIRE and co-occurrence in the same metagenome. The scale bar represents substitutions per site. Branches are collapsed if support was low (< 50), circles indicate moderate support (50–80, pale pink) or high support (80–97, green), branches without circles are fully supported (> 97). Tree is rooted at the poxviruses. The blue polygon indicates virophage-host linkage as predicted in **c**. **c** Schematic of the analysis of 699 marine microbial eukaryotic transcriptome sequencing projects (MMETSP). In the transcriptome of *Alexandrium tamarense*, the virophage four core genes were identified in two separate contigs (one containing the MCP and the other one the remaining three core genes) as well as a giant viral MCP and a DNA polymerase type

Here, we predicted co-infecting viruses and their eukaryotic hosts based on a recently proposed virophage resistance mechanism, the *Mimivirus* virophage resistance element (MIMIVIRE) system. This mechanism identified in *A. polyphaga* mimivirus (APMV), includes a specific sequence shared between the virophage and its associated giant virus, which is present in multiple copies in the giant virus genome [36]. This observation led to the proposal of a mechanism whereby the translation of the sequence insertion responded to a protein-based interaction model where a *Mimivirus* protein might inhibit the function of the virophage by competing for resources to generate the same peptidic motifs [37]. We thus searched for amino acid patterns shared (Fig. 5a) between any of the predicted virophage sequences (> 10 kb) and giant viruses from an in-house nucleocytoplasmic large DNA viral (NCLDV) database (see the “Methods” section). We found seven connections between giant viral contigs with predicted taxonomy and virophages (Fig. 5a, b). The taxonomy of these giant viruses was inferred based on a concatenated alignment of five core nucleocytoplasmic virus orthologous genes (NCVOGs) [38] (see the “Methods” section). We observed that most giant viruses were affiliated with the *Mimiviridae*, with the majority branching within the *Mesomimivirinae* subfamily. This result is consistent with previous reports where co-infecting giant viruses have usually been members of *Megamimivirinae* or the genus *Cafeteriavirus* (e.g., APMV and CroV) (Fig. 5b). For one of our predicted virophage-NCLDV associations, the two viruses were found in the same lake sample (Fig. 5b). There was no sharing of protein content between members of virophage-NCLDV pairs, although this analysis was limited by the fact that most giant virus genomes are incomplete. Besides *Mimiviruses*, we predicted one virophage to be associated with a virus from the *Asfar-Faustovirus* cluster. While *Asfarviruses* are known to infect insects and swine, *Faustoviruses* infect amoebae [39]. This would be the first case of a giant virus from this group connected with a virophage genome. We also attempted to identify eukaryotic hosts for co-infecting virophage and giant viruses by searching for their sequences in publicly available marine microbial eukaryote transcriptomes [40] (Fig. 5c). We found two virophages associated with two marine protists. One virophage was detected in *Bigelowiella natans*, a chlorarachniophyte alga that is a model organism for the Rhizaria [41]. The *B. natans* virophage sequence found in this study was previously described as a provirophage [42] integrated into the algal chromosome. The second virophage was identified in two separate contigs (one containing the MCP and the other one the remaining three core genes) in the transcriptome of the dinoflagellate *Alexandrium tamarense*. In this transcriptome, we also identified one giant virus MCP. We extracted all contigs with hits to conserved NCLDV marker genes (see the “Methods” section) and predicted that this sample had a single giant virus that was closely related to CroV (Fig. 5b). This giant virus has been previously reported to co-infect along with *Mavirus*, a virophage very closely related to the novel *A. tamarense* virophage (clade 1 and clade 2, respectively) (Fig. 3). However, the eukaryotic host of *Mavirus*, *Cafeteria roenbergensis*, is a member of the phylum Heterokontophyta, which is distantly related to the phylum Dinoflagellata that includes *A. tamarense*, suggesting that related virophages and giant viruses may infect very distant eukaryotic hosts.

## Discussion

Virophages are recently discovered viral entities that require giant viruses to co-infect eukaryotic microbes. Their complex interactions make them very difficult to isolate in the laboratory and there are only a few isolated representatives derived from co-culture experiments. To bypass the hurdles of experimental identification of virophages and explore the range of their phylogenetic and habitat diversity, we developed a computational approach harnessing the information available in over 14,000 metagenomic samples. Our approach relied on the availability of a unique and conserved virophage signature gene encoding for the major capsid protein (MCP). Through an iterative process, MCP-specific HMM models were developed leading to the identification and characterization of hundreds of high-quality (HQ) virophage genomes across a large diversity of habitats. Although the results might be biased due to the overrepresentation of MCPs from published virophages found in aquatic habitats and the metadata of the samples from the databases analyzed (e.g., habitat distribution and sequencing/assembly technology used), the global survey of virophages enabled by this approach can lead to a better understanding of virophage biology, habitat diversity, taxonomy, and evolution.

Before this work, only 33 HQ virophage genomes from both isolates and metagenome-derived genomes were identified and classified as members of the *Lavidaviridae* family. Below the family level, virophage classification relied on the presence of “at least some of the morphogenetic genes conserved in virophages (MCP, mCP, ATPase, PRO)” and “the dependency or association of the virus with a NCLDV.” This classification resulted in two separate genera (genus *Sputnikvirus* and genus *Mavirus*) [10]. In addition, it was proposed that other known metagenome-derived virophages (OLV, YSLVs, and rumen virophages) were likely to be classified in different genera, but the absence of replicating isolates limited their classification by the ICTV. Biogeography studies have previously used partial MCPs from known virophages for homology-based searches to propose a global distribution across microbiomes [13]. However, the identification of HQ virophage genomes has been very limited and biased towards aquatic environments [13, 15–17].

This study revealed that the vast majority of the virophage protein clusters (VpPC) were shared by less than 5% of the genomes, indicating an enormous genetic diversity which could be attributed to the virophage evolutionary position and high frequency of horizontal gene exchange with other viral entities and microbial cells [43]. However, the previously proposed four core gene families were present among all the newly identified complete genomes, including the ruminant-associated virophage genomes where the mCP was previously reported as missing [18]. This finding is essential for the new classification scheme proposed for microbiome-derived HQ virophages that were based on sequence homology and gene synteny of the conserved VpPCs. Our approach revealed that 17 out of the 27 proposed clades are novel, while the remaining 10 (associated with published virophages and in agreement with the previous classification) were vastly expanded with new sequences. This classification was further supported by the MCP type, the habitat type distribution, and the overall gene content of the clade members (Fig. 3) and revealed a great increase in the diversity of the different taxonomic groups defined by HQ virophage genome sequences.

Freshwater samples continued to be the habitat with the largest number of virophages recovered and still the reservoirs with the largest number of MCP sequences in clades without HQ genomes. As an example, 80% and 75% of the virophages from the clades 19 and 24 (764 and 2455 MCP members, respectively) were recovered from freshwater samples (Fig. 2a). In addition, for the first time, we found HQ virophage genomes in other diverse habitats including plant-associated, thermal springs, deep-subsurface, cow rumen, and human-gut samples. Particularly interesting was the case of the human gut-associated virophages, which were characterized by quite distinct MCP models (Fig. 4c). Four out of the five human-associated HQ virophage genomes were identified in fecal samples recovered from individuals with a rural lifestyle, with the remaining genome found in an individual with ulcerative colitis. Accordingly, these virophages could be connected to the intake of unicellular eukaryotes with food or water. This observation was also supported by the distribution of the MCP models found in fecal samples from individuals with rural lifestyle, which were shared primarily with animals (baboon, cow, sheep, and arthropods) and freshwater sources (Fig. 2c).

Despite the tremendous variability of protein content encoded by the predicted virophage genomes, this lineage is characterized by the presence of a syntenic block of 4–5 genes found in multiple genomes from distant parts of the virophage tree suggesting that these genes were vertically inherited from a common ancestor. However, the variation of synteny within this block between proposed virophage clades is indicative of significant genome reorganization.

A number of VpPCs (e.g., integrases, methylases, recombinases, and DNA polymerases) have homologs in viruses outside of the virophage lineage, especially in polintons and polinton-like viruses. This suggests frequent gene transfers between these different types of mobile genetic elements, as previously hypothesized [22, 44]. This was also supported by phylogenies of the DNA polymerase type B and rve integrase showing mixed clades gathering virophages, polintons, and polinton-like viruses (Additional file 2: Figure S2). From this pool of genes, of particular interest is the presence of integrases, recombinases, and transfer RNAs in virophages. Integrases and recombinases were identified across the majority of the proposed virophage clades (Additional file 1: Table S4; Additional file 1: Table S5), likely providing those viruses with the ability to incorporate their DNA into the host genome as provirophages. Integration was previously described for *Mavirus* and *Bigelowiella natans* virophages [7, 42, 45] and could provide potential protection for the eukaryotic host against NCLDVs [42]. On the other hand, this is the first time that tRNA sequences were identified in virophage genomes (Additional file 2: Figure S6). Their presence might help virophages to complement their host’s codon or amino acid usage [32, 33] or could be a result of acquisition from the host genome since tRNAs are known as hot spots for virus integration [32, 34, 35].

Finally, a novel MIMIVIRE-based computational approach to predict the association of virophages with giant viruses revealed novel giant virus lineages potentially targeted by virophages. In addition, the analysis of protozoan transcriptomes enabled the detection of the triple association between a *Mavirus*-related virophage, a CroV-related giant virus, and a marine dinoflagellate *A. tamarense*. We anticipate that these data will drive further experimental design and validation of the computational predictions of virophage-giant virus-microeukaryote triplets and elucidate the evolution and ecology of these remarkable biological systems.

## Methods

### Development of major capsid protein models

Hidden Markov models (HMMs) were built from sequences of the major capsid protein through a two-step process and were used to interrogate public microbiomes. First, the major capsid protein (MCP) sequences of all the published virophages as of August 2017 were identified (from Bekliz et al. [11]) and used to search for homologs across the IMG/VR virus database [24] using the blastp program from the BLAST+ package [46] with an *e* value cutoff of 1 × 10^−06^. This led to the detection of 84 virophage-MCP-like genes recovered from 80 metagenomic viral contigs. We then clustered the total set of MCP genes (published and newly discovered metagenome-derived) with bidirectional cutoffs (> = 30% identity over > = 70% alignment fraction) after alignment (ClustalOmega algorithm [47]) using the Markov clustering (MCL) [48]. Four MCP families (models) were created using *hmmbuild* from the *hmmer* v3.1b2 package [49]. We compared these models against all assembled metagenomic contigs from the Integrated Microbial Genomes with Microbiome Samples (IMG/M) system [25] and identified 35,304 unique sequences with hits to the models (*e* value < 1e−06). We used the 9813 newly identified MCP sequences larger than 700 nt to complement the MCP sequences from the published virophages. We repeated the steps described above (de-replication using blast 30–70%, Clustal Omega alignment, MCL clustering, and *hmmbuild*). We created 15 clusters (all of them with at least 60 members).

### Screening metagenomes and identification of virophage genomes

The 15 MCP models were used to interrogate > 10,000 public microbiomes from the IMG/M system (over 5 Tb of assembled metagenomic sequence data [25] where samples from host-associated, terrestrial, engineered, marine, freshwater, non-marine saline, thermal vents, sediments, and air habitats, representing the 34%, 17%, 16%, 15%, 11%, 2%, 2%, 2%, and 1% of the total set, respectively, are included) and 3771 human gut assembled samples [50] from the sequence read archive (SRA, https://www.ncbi.nlm.nih.gov/sra) (details below). We used the *hmmsearch* tool from the *hmmer* v3.1b2 package [49] to identify unique sequences with hits to the models (*e* value < 1e−06) and identified 44,221 metagenome-derived complete and partial MCPs. In order to identify unique MCP sequences and reduce the redundancy, we de-replicated the MCPs using a cutoff of 95% sequence identity over 95% coverage of the length of the shortest sequence. This process resulted in a final list of 28,294 unique MCP sequences that were used to infer the global habitat distribution of the virophages. We used the amino acid average size of the published MCPs (593 aa) +/− 1 standard deviation (+/− 40.1) to estimate the completeness of the MCP gene and predicted that 4% of the sequences were complete and 11% over 50% of the predicted size. We then recovered 477 virophage contigs larger than 10 kb (after a de-replication process based on 95% identity over 80% of the length on the shortest contig; Additional file 1: Table S3) from geographically and ecologically diverse samples from which 70% of them (328) contained the set of four “core” genes and were referred as high-quality (HQ) virophages. Complete virophage genomes can be circular [5] or linear with inverted terminal repeats (ITR) [18]. Circularity was detected based on overlapping 5′ and 3′ ends, and ITR of at least 100 bp were searched for linear contigs.

### Human gut NCBI SRA samples

Three thousand seven hundred seventy-one human fecal metagenomes were downloaded from the NCBI SRA and assembled using MegaHIT v1.1.1 [51] using default parameters. These datasets included samples from a wide range of countries, age groups, and disease states [50]. Protein coding genes were identified from metagenomic contigs using Prodigal v2.6.3 [52] with default parameters. *Hmmer* v3.1b2 was used to search identified proteins against the database of 15 virophage marker genes using the *hmmsearch* program [49] with default parameters. Homologs were identified with *e* values < 1e−06.

### Phylogenetic analysis of conserved virophage genetic loci

Predicted amino acid sequences of all the virophage full-length four core genes (MCP, mCP, ATPase, and PRO) were aligned using MAFFT (version 7) with default parameters [53]. The alignments of each gene were concatenated and then trimmed using trimAL (version 1.2) with the option “-gappyout” [54]. The trimmed concatenated alignment was used as input into Fasttree 2.1 to reconstruct a maximum-likelihood phylogenetic tree with 1000 iterations using a substitution model of WAG. This tree was then inputted into the interactive tree of life (iTOL) software [55] to add information on the MCP HMM model, habitat type, gene synteny, genome length, and structure. Branches were auto-collapsed using an average branch length distance < 1.2 substitutions per site followed by manual adjustment for a good match to the core-gene syntenies and classified into biologically significant phylogenetic groups named “clades”. Bootstrap confidence levels in all collapsed clades were greater than 0.8. Alignments and tree construction for the type B DNA polymerases and rve integrases followed the same steps: alignment using MAFFT (version 7), followed by the tree construction using Fasttree 2.1 [56] with a substitution model of WAG.

### Clustering of virophage proteins and genome annotation

A set of 10,064 proteins predicted from the new virophage contigs (477 sequences ≥ 10 kb) were clustered along with proteins predicted from 56 reference genomes. These references included previously published virophage genomes that were (1) sequenced from isolates [5, 7–9, 57], (2) assembled from metagenomes [12–17], or (3) detected in protist genomes [42]. Sequences from polinton viruses were also included [58]. A two-step clustering, similar to that performed in [16], was computed as follows. Protein sequences were first compared using blastp (all-vs-all comparison, BLAST*+* v2.6.0, threshold of 30 on score and 0.01 on *e* value). This set of BLAST hits defined a weighted network in which predicted proteins were nodes, and edges were connections between these predicted proteins with a weight proportional to the hit score. Groups of similar proteins were detected on this network using the InfoMap tool (two-level hierarchy, default parameters otherwise [59]). Next, a profile analysis was computed to gather these groups into larger clusters of homologous sequences (hereafter “protein clusters”, or “PCs”), using tools from the HH-suite package [49]. Sequences in each group were first clustered at 90% identity with *cd-hit* [60], aligned with muscle [61], and a profile was built with hhmake [62]. Profile-profile comparisons were computed using HHSearch (parameters: -M 50 -norealign -nocons -nopred -nodssp -E 0.001, [63]). Hits between profiles were selected based on their probability, coverage, and length: all hits with probability ≥ 90% and coverage ≥ 50% were selected, as well as hits with probability ≥ 99%, coverage ≥ 20%, and length ≥ 100 amino acids. These parameters were selected based on the grouping of four virophage core genes in single PCs, as in [16]. This approach yielded 711 PCs (i.e., groups of 2 or more proteins), encompassing 7810 predicted proteins in total.

### Nucleocytoplasmic large DNA virus (NCLDV) database

Similar to the virophage MCP HMM development, we built a giant virus MCP model that allowed the identification of NCLDV contigs from microbiomes. Metagenomic sequences from the IMG/M system with homology (blastp program from the BLAST+ package [46] with an *e* value cutoff of 1 × 10^−06^) to the major capsid proteins (MCP) of reference *Mimiviruses* were used to detect 544 NCLDV-MCP-like genes. Then, the total set of MCP genes (reference and metagenome-derived) were clustered with bidirectional cutoffs (> = 30% identity over > = 70% alignment fraction) after alignment (ClustalOmega algorithm [47]) using the Markov clustering (MCL) [48]. One MCP model was created using *hmmbuild* from the *hmmer* v3.1b2 package [49]. We compared this model against all assembled metagenomic contigs from the IMG/M system [25] and identified 17,551 unique sequences with hits (*e* value < 1e−06) and larger than 5000 bp as members of this database.

### Virophage-giant virus connection via the MIMIVIRE system

Virophage and giant viral contigs were connected when they shared at least one sequence (at 100% identity) of 24–30 nt in both genomes and at least one repeated subset (~ 18 nt) of the shared sequence within the same giant viral gene [36]. The sequences were also translated to determine if the shared region also contained the same amino acid frameshift.

### Nucleocytoplasmic large DNA virus (NCLDV) phylogenomics

As a backbone for phylogenetic and shared protein content analyses, 184 NCLDV genomes available at NCBI Genbank were downloaded and clustered at an average nucleotide identity (ANI) of 95% with fastANI [64], resulting in 116 clusters. To infer the phylogenetic positions of the metagenomics NCLDV contigs, five core NCLDV proteins [38] were selected: DNA polymerase elongation subunit family B (NCVOG0038), D5-like helicase-primase (NCVOG0023), packaging ATPase (NCVOG0249) and DNA or RNA helicases of superfamily II (NCVOG0076), poxvirus late transcription factor VLTF3-like (NCVOG0262), and identified with *hmmsearch* (*hmmer* version 3.1b2). Reference genomes and metagenomic contigs with at least three out of five marker proteins were included in the analysis. Protein sequences were aligned with *MAFFT* [65]; gapped columns in alignments (more than 90% of gaps) were removed with trimal [54]. A phylogenetic tree was built from the concatenated alignment of all five proteins using *IQ-tree* with LG + F + R6 [66]. Protein families were inferred with *OrthoFinder* 1.03 [67] with default settings from a representative dataset of 116 NCLDV genomes and 12 metagenomic NCLDV contigs.

## Conclusions

In conclusion, we present a global metagenomic study of virophages using a computational approach resulting in the identification of 328 new high-quality genomes and over 45,000 virophage genome fragments. This represents a massive increase compared to previously known virophages that allowed us to conduct in-depth analysis of their genomes confirming previous results from others (i.e., presence of the four core genes) and drawing novel biological conclusions (e.g., ancient synteny of the four core genes, discovery of high-quality virophage genomes from unreported habitats including human gut, revised virophage classification, prediction of eukaryotic virus hosts for several virophages, and degree of genome mobility) about these important entities of the viral world. Overall, we provide a global analysis of the diversity, distribution, and evolution of virophages.

## Supplementary information

**Additional file 1.** Supplementary tables (XLS 11079 kb)

**Additional file 2.** Supplementary data

## Data Availability

MCP HMM models and HQ Metagenomic virophage sequences are available on the JGI FTP site http://portal.nersc.gov/dna/microbial/prokpubs/virophage. Assembled sequences for virophage MCPs, NCLDV genomes, and eukaryotic contigs are available at the IMG/M public system using the taxon and scaffold identifiers provided alongside the article and tables.

## Abbreviations

ALM: *Acid Lake Mavirus*
APMV: *Acanthamoeba polyphaga* mimivirus
CroV: *Cafeteria roenbergensis* virus
DNApolB: Type B DNA polymerase
HMM: Hidden Markov motif
HQ virophage: High-quality virophage genome
IMG/M: Integrated Microbial Genomes with Microbiomes
IMG/VR: Integrated Microbial Genomes and Microbiomes with Virus
ITR: Inverted terminal repeat
MCP: Major capsid protein
mCP: Minor capsid protein
MIMIVIRE: *Mimivirus* virophage resistance element
MMETSP: Marine microbial eukaryotic transcriptome sequencing projects
MTase: Methyl transferase
NCLDV: Nucleocytoplasmic large DNA viruses
NCVOG: Clusters of orthologous genes for NCLDV genomes
PRO: Cysteine protease
rve-INT: Retroviral integrase
tRNA: Transfer ribonucleic acid
Vp.cl: Virophage clade
VpPC: Virophage protein cluster
