# Supplementary material for: Diversity, evolution, and classification of virophages uncovered through global metagenomics
Source: Microbiome. 2019 Dec 10;7:157. doi: 10.1186/s40168-019-0768-5 (PMC6905037; doi:10.1186/s40168-019-0768-5)
Supplement: Supplementary file 2 — Additional file 2. Supplementary data [file 40168_2019_768_MOESM2_ESM.zip › SFig_S1.pdf]

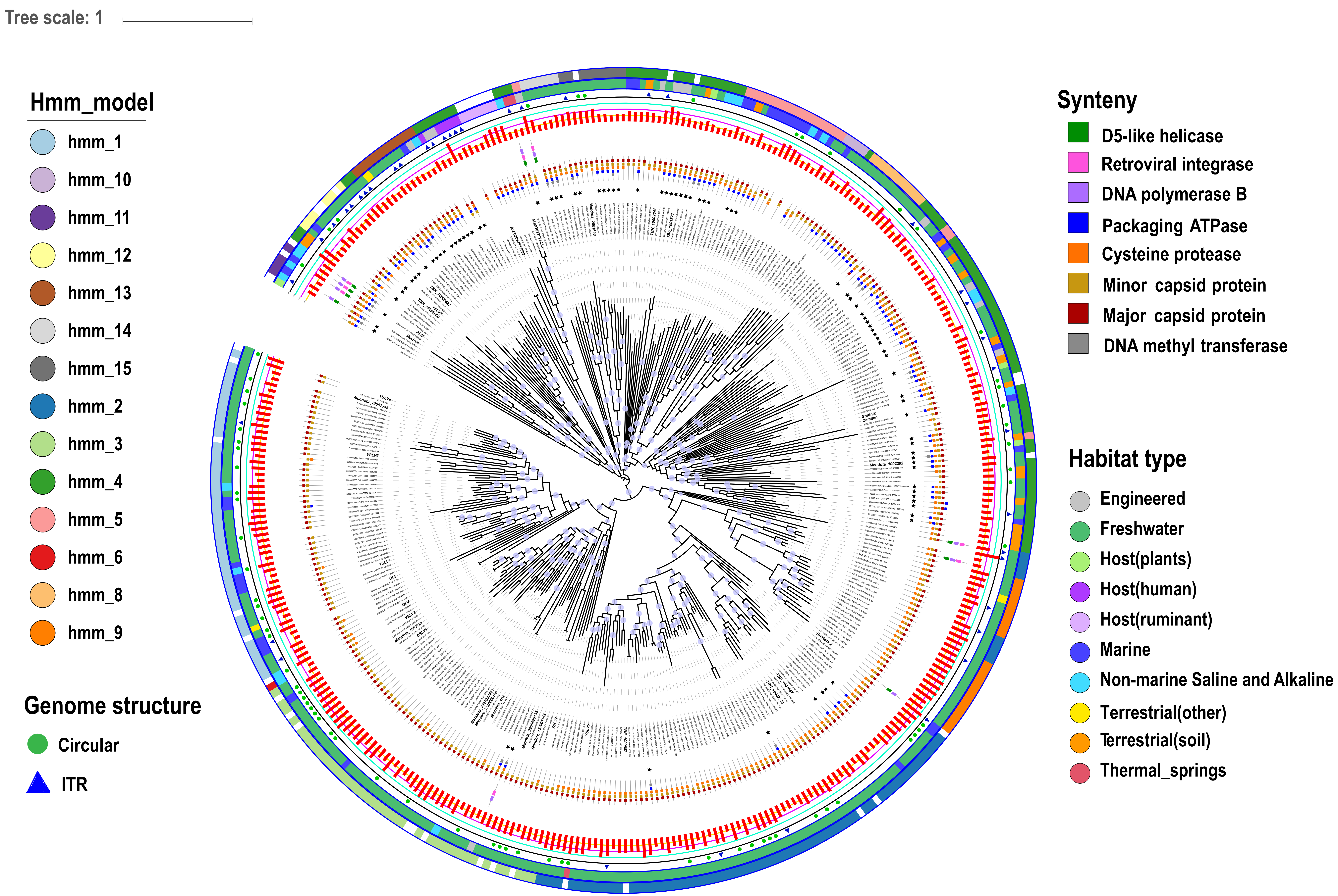

Tree scale: 1
Hmm_model
hmm_1
hmm_10
hmm_11
hmm_12
hmm_13
hmm_14
hmm_15
hmm_2
hmm_3
hmm_4
hmm_5
hmm_6
hmm_8
hmm_9
Genome structure
Circular
ITR
Synteny
D5-like helicase
Retroviral integrase
DNA polymerase B
Packaging ATPase
Cysteine protease
Minor capsid protein
Major capsid protein
DNA methyl transferase
Habitat type
Engineered
Freshwater
Host(plants)
Host(human)
Host(ruminant)
Marine
Non-marine Saline and Alkaline
Terrestrial(other)
Terrestrial(soil)
Thermal_springs
