# Supplementary material for: Diversity, evolution, and classification of virophages uncovered through global metagenomics
Source: Microbiome. 2019 Dec 10;7:157. doi: 10.1186/s40168-019-0768-5 (PMC6905037; doi:10.1186/s40168-019-0768-5)
Supplement: Supplementary file 2 — Additional file 2. Supplementary data [file 40168_2019_768_MOESM2_ESM.zip › SFig_S5.pdf]

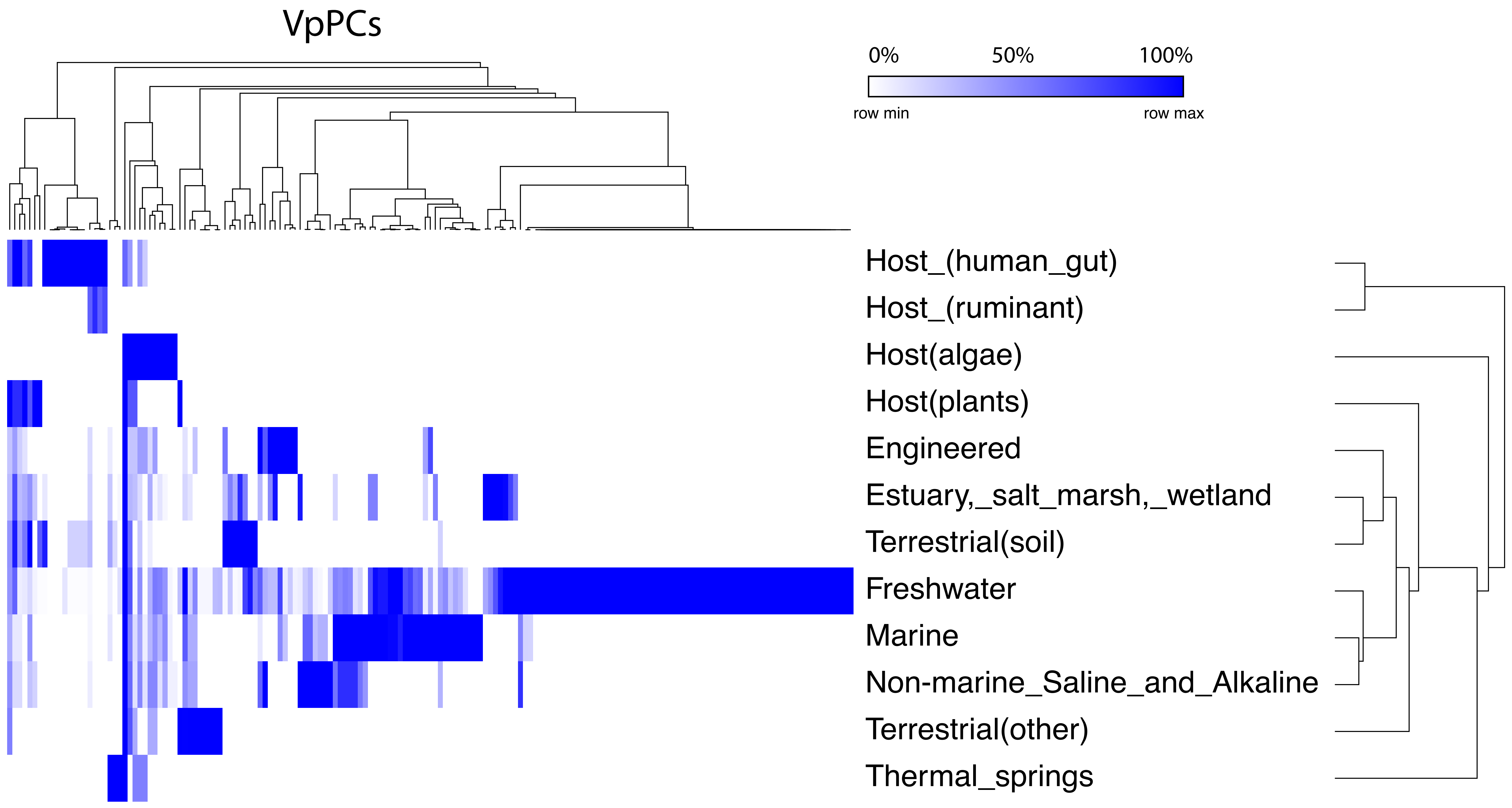
VpPCs
0%
50%
100%
row min
row max
Host_(human_gut)
Host_(ruminant)
Host(algae)
Host(plants)
Engineered
Estuary,_salt_marsh,_wetland
Terrestrial(soil)
Freshwater
Marine
Non-marine_Saline_and_Alkaline
Terrestrial(other)
Thermal_springs
