# Supplementary material for: Diversity, evolution, and classification of virophages uncovered through global metagenomics
Source: Microbiome. 2019 Dec 10;7:157. doi: 10.1186/s40168-019-0768-5 (PMC6905037; doi:10.1186/s40168-019-0768-5)
Supplement: Supplementary file 2 — Additional file 2. Supplementary data [file 40168_2019_768_MOESM2_ESM.zip › SFig_S6_A.pdf]

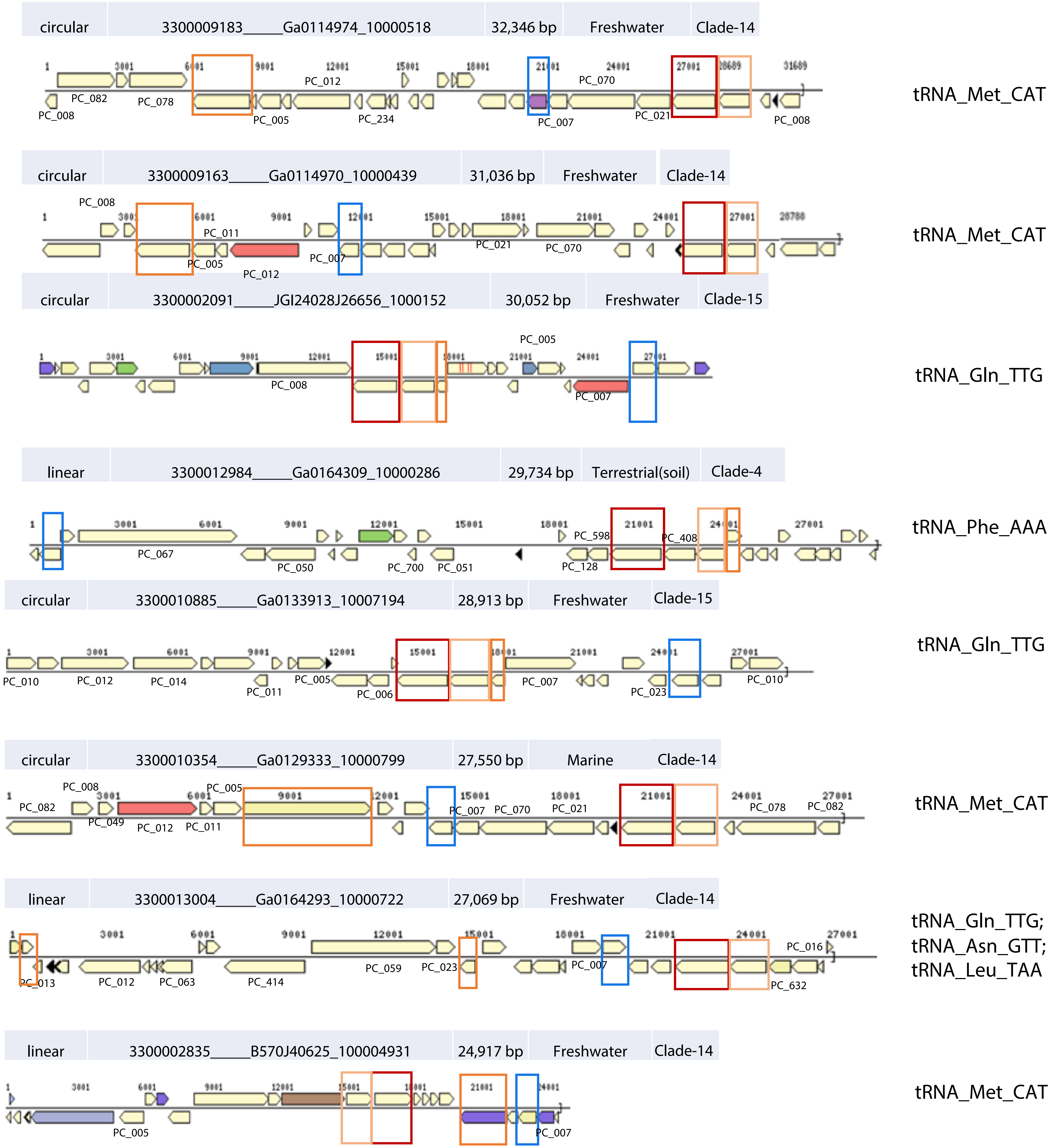
circular
3300009183_____Ga0114974_10000518
32,346 bp
Freshwater
Clade-14
PC_082
PC_078
PC_008
PC_012
PC_005
PC_234
PC_070
PC_007
PC_021
PC_008
tRNA_Met_CAT
circular
3300009163_____Ga0114970_10000439
31,036 bp
Freshwater
Clade-14
PC_008
PC_011
PC_005
PC_012
PC_007
PC_021
PC_070
tRNA_Met_CAT
circular
3300002091_____JGI24028J26656_1000152
30,052 bp
Freshwater
Clade-15
PC_005
PC_008
PC_007
tRNA_Gln_TTG
linear
3300012984_____Ga0164309_10000286
29,734 bp
Terrestrial(soil)
Clade-4
PC_067
PC_050
PC_700
PC_051
PC_598
PC_128
PC_408
tRNA_Phe_AAA
circular
3300010885_____Ga0133913_10007194
28,913 bp
Freshwater
Clade-15
PC_010
PC_012
PC_014
PC_011
PC_005
PC_006
PC_007
PC_023
PC_010
tRNA_Gln_TTG
circular
3300010354_____Ga0129333_10000799
27,550 bp
Marine
Clade-14
PC_008
PC_082
PC_049
PC_012
PC_005
PC_011
PC_007
PC_070
PC_021
PC_078
PC_082
tRNA_Met_CAT
linear
3300013004_____Ga0164293_10000722
27,069 bp
Freshwater
Clade-14
PC_013
PC_012
PC_063
PC_414
PC_059
PC_023
PC_007
PC_016
PC_632
tRNA_Gln_TTG;
tRNA_Asn_GTT;
tRNA_Leu_TAA
linear
3300002835_____B570J40625_100004931
24,917 bp
Freshwater
Clade-14
PC_005
PC_007
tRNA_Met_CAT
