# Supplementary figures and images for: Diversity, evolution, and classification of virophages uncovered through global metagenomics
Source: Microbiome. 2019 Dec 10;7:157. doi: 10.1186/s40168-019-0768-5 (PMC6905037; doi:10.1186/s40168-019-0768-5)

(A) DNA polymerase type B (DNApolB)

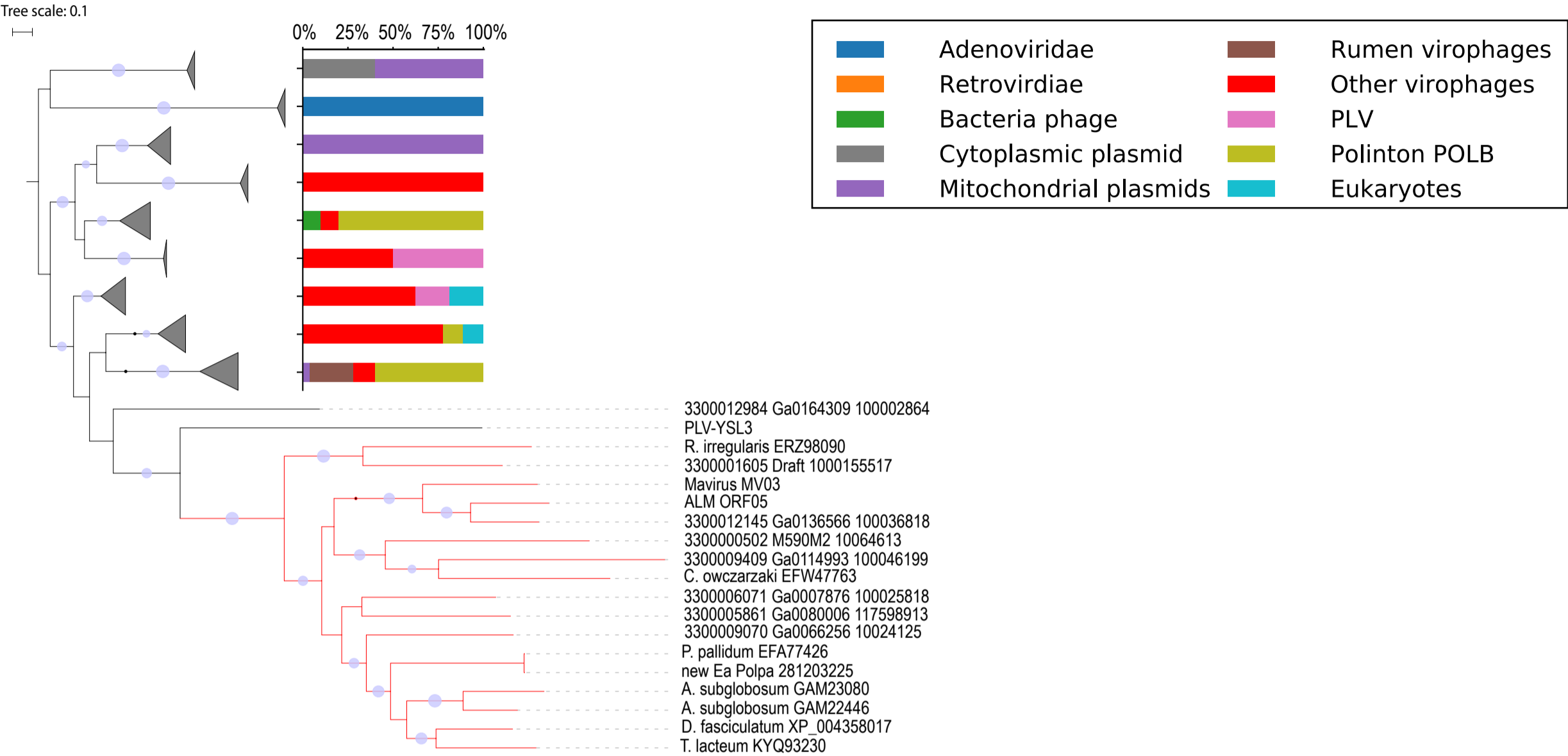

(B) Retroviral integrase (rve INT)

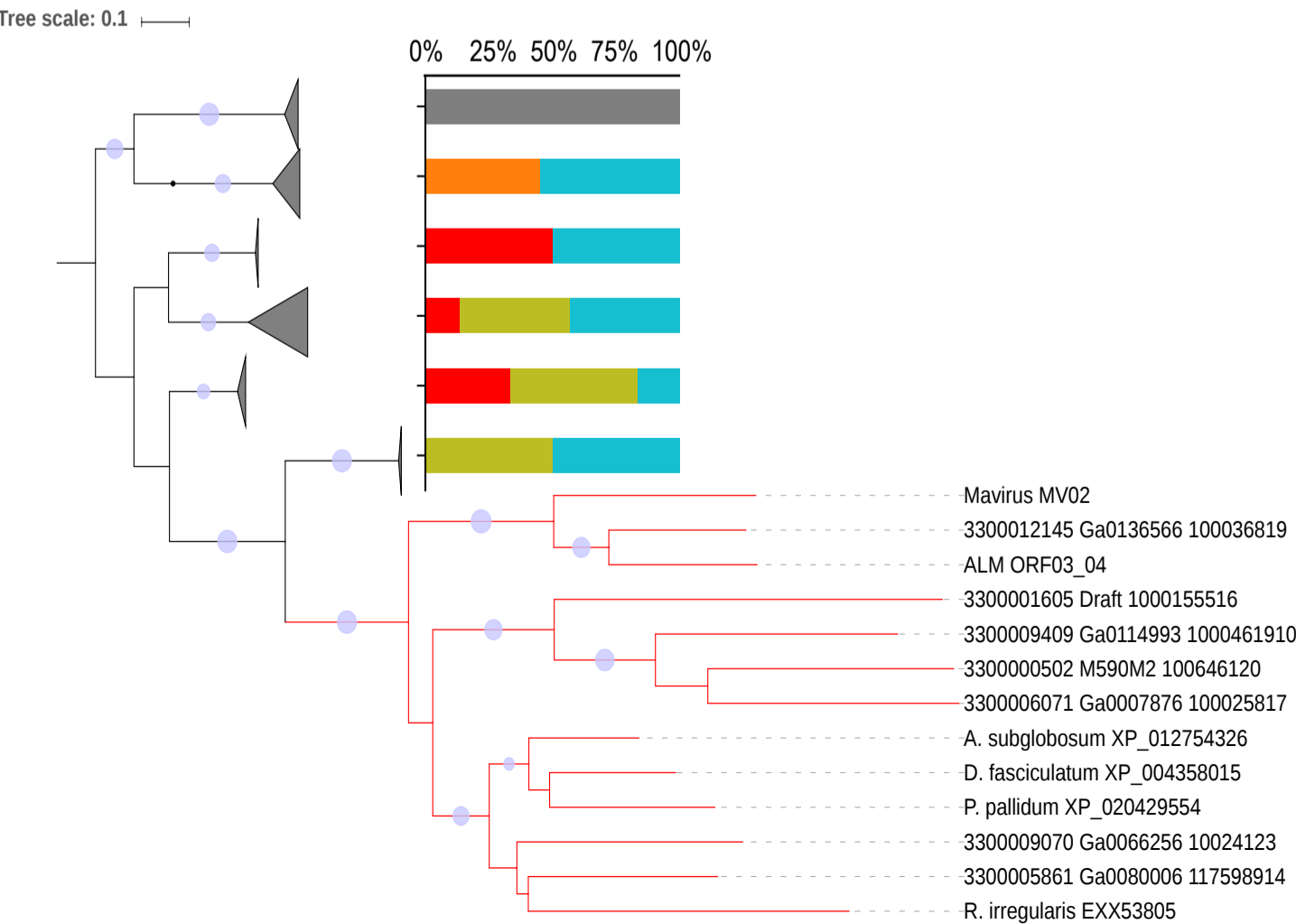

Supplement: Supplementary file 2 — Additional file 2. Supplementary data [file 40168_2019_768_MOESM2_ESM.zip › SFig_S2.pdf]

Virophage protein clusters: Number of members

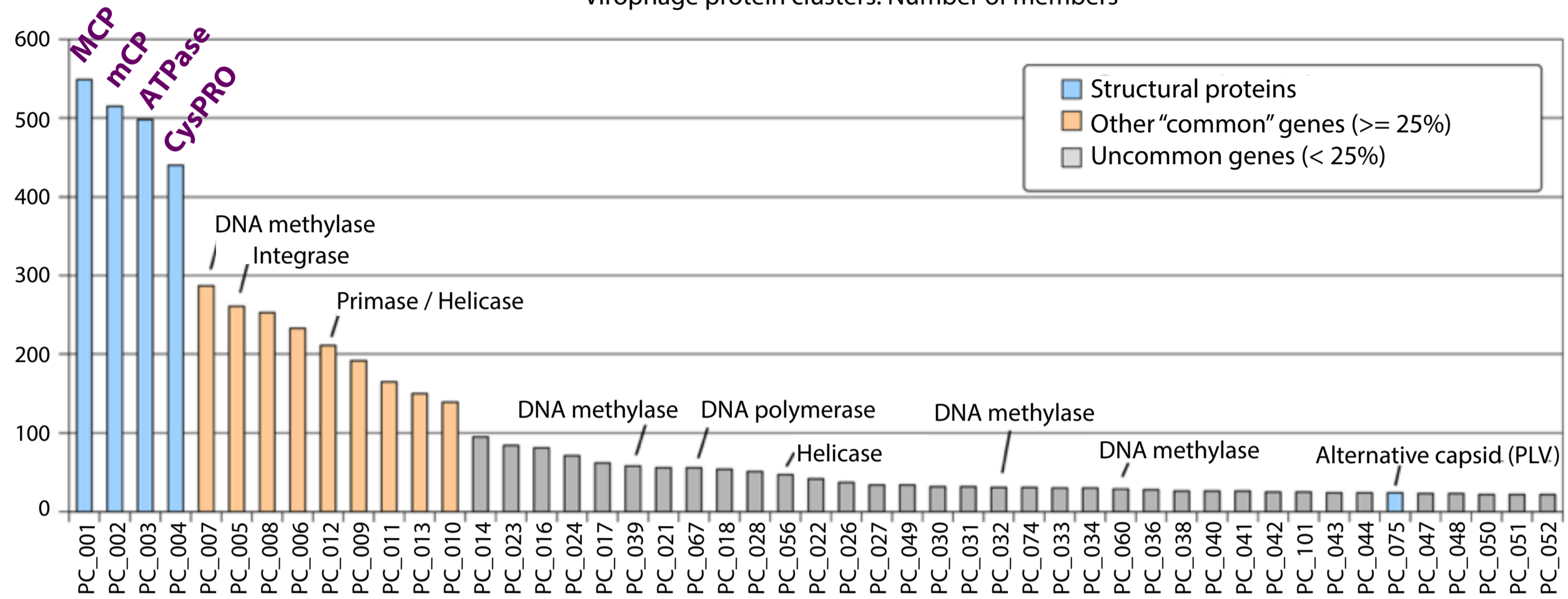

Supplement: Supplementary file 2 — Additional file 2. Supplementary data [file 40168_2019_768_MOESM2_ESM.zip › SFig_S3.pdf]

VpPCs

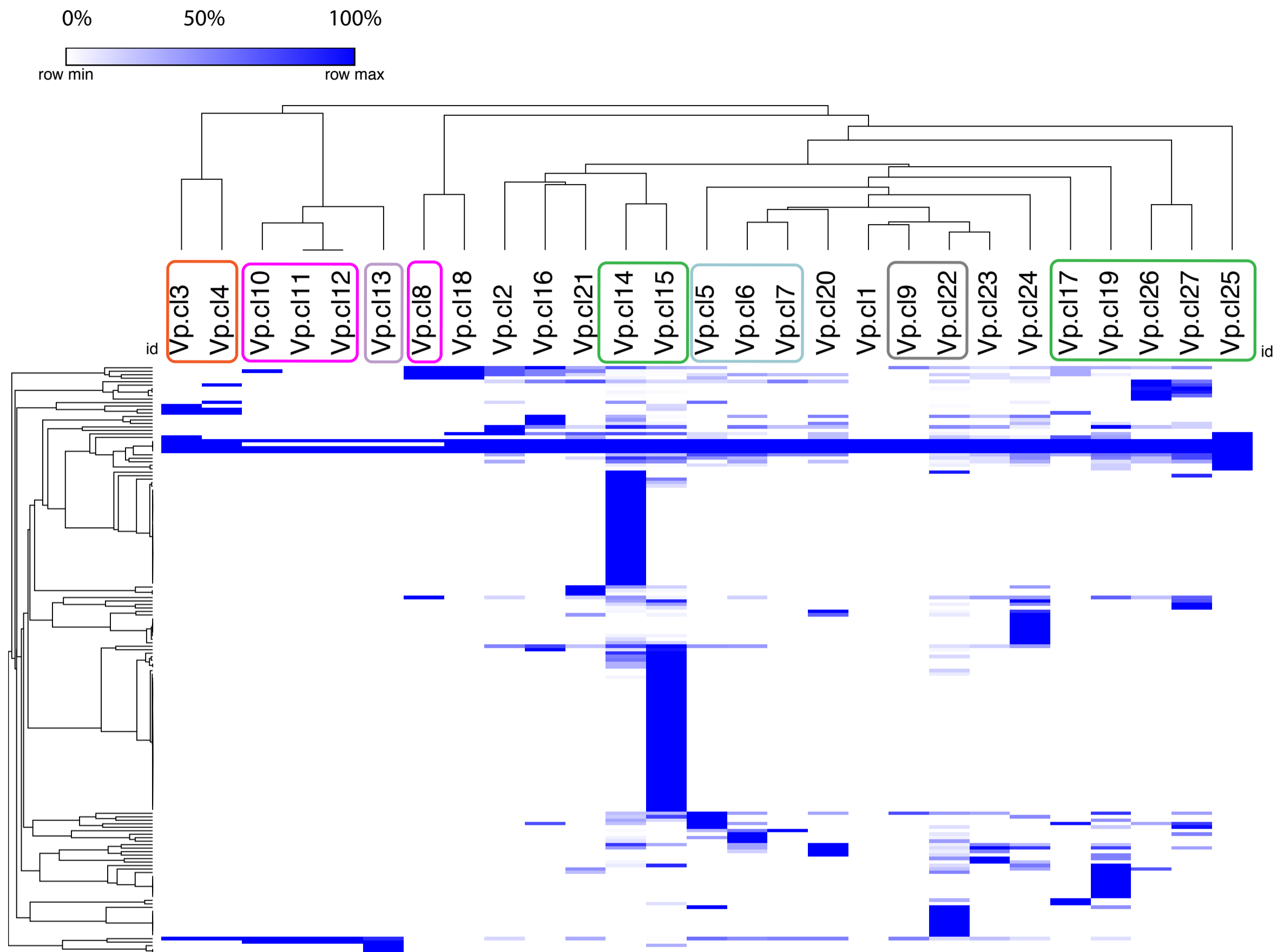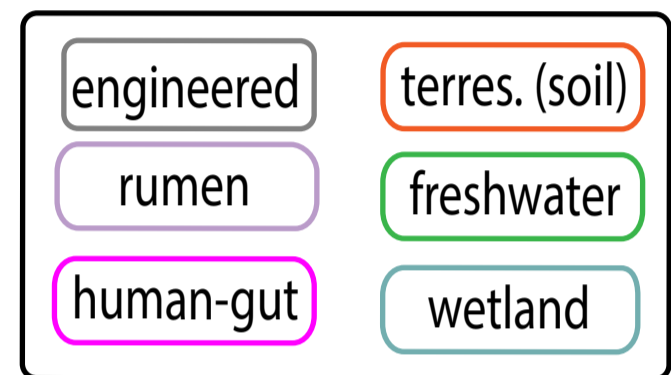

Supplement: Supplementary file 2 — Additional file 2. Supplementary data [file 40168_2019_768_MOESM2_ESM.zip › SFig_S4.pdf]
