# Supplementary material for: Diversity, evolution, and classification of virophages uncovered through global metagenomics
Source: Microbiome. 2019 Dec 10;7:157. doi: 10.1186/s40168-019-0768-5 (PMC6905037; doi:10.1186/s40168-019-0768-5)
Supplement: Supplementary file 2 — Additional file 2. Supplementary data [file SFig_S6_B.pdf › SFig_S6_B.pdf]

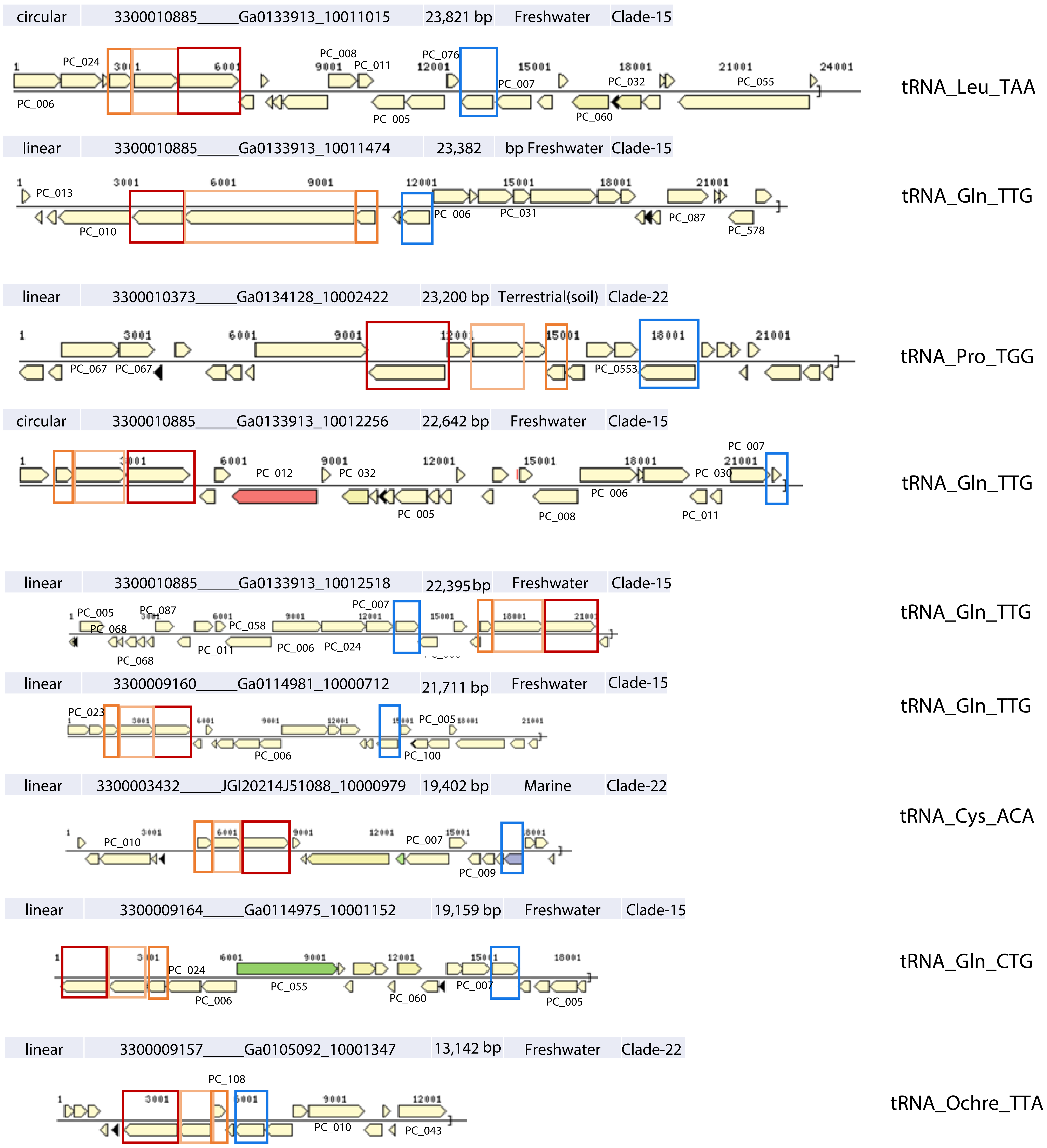

circular 3300010885_____Ga0133913_10011015 23,821 bp Freshwater Clade-15
PC_024 PC_006 PC_008 PC_011 PC_076 PC_005 PC_007 PC_032 PC_060 PC_055
tRNA_Leu_TAA
linear 3300010885_____Ga0133913_10011474 23,382 bp Freshwater Clade-15
PC_013 PC_010 PC_006 PC_031 PC_087 PC_578
tRNA_Gln_TTG
linear 3300010373_____Ga0134128_10002422 23,200 bp Terrestrial(soil) Clade-22
PC_067 PC_067 PC_0553
tRNA_Pro_TGG
circular 3300010885_____Ga0133913_10012256 22,642 bp Freshwater Clade-15
PC_012 PC_032 PC_005 PC_008 PC_006 PC_007 PC_030 PC_011
tRNA_Gln_TTG
linear 3300010885_____Ga0133913_10012518 22,395 bp Freshwater Clade-15
PC_005 PC_087 PC_068 PC_058 PC_007 PC_068 PC_011 PC_006 PC_024
tRNA_Gln_TTG
linear 3300009160_____Ga0114981_10000712 21,711 bp Freshwater Clade-15
PC_023 PC_005 PC_006 PC_100
tRNA_Gln_TTG
linear 3300003432_____JGI20214J51088_10000979 19,402 bp Marine Clade-22
PC_010 PC_007 PC_009
tRNA_Cys_ACA
linear 3300009164_____Ga0114975_10001152 19,159 bp Freshwater Clade-15
PC_024 PC_006 PC_055 PC_060 PC_007 PC_005
tRNA_Gln_CTG
linear 3300009157_____Ga0105092_10001347 13,142 bp Freshwater Clade-22
PC_108 PC_010 PC_043
tRNA_Ochre_TTA
